# Consequences of asynchronous heading in a perennial bunchgrass (*Elymus excelsus*)

**DOI:** 10.1038/s41598-018-21759-2

**Published:** 2018-02-20

**Authors:** Haiyan Li, Xiaowei Jin, Yunfei Yang

**Affiliations:** Key Laboratory of Vegetation Ecology, Ministry of Education, Institute of Grassland Science, Northeast Normal University, Changchun, Jilin Province P. R. China

## Abstract

Reproduction is vital to plant population adaptation. The consequences of asynchronous reproduction in a perennial bunchgrass grass is not well studied. The heading reproductive tillers from early to late forms a continuum due to asynchronous heading and flowering in *Elymus excelsus* population. In two peak years of production, the three-year-old and four-year-old reproductive tillers of experimental *E. excelsus* population were marked from the early to late heading stage every four days at five different heading times and these tillers were harvested at the dough stage, respectively. The growth, biomass, seed production and reproductive allocation were measured to analyze the consequences of asynchronous reproduction. Reproductive tiller height, biomass, inflorescence length, inflorescence biomass, floret number, seed number, seed biomass, seed-set percentage, biomass allocation to inflorescence (RA1) and to seed (RA2) significantly decreased with the delay of heading date over the two years. Above ten phenotypic characteristics exponentially increased at a significant or extremely significant level with increasing differences in reproductive period. Reproductive tillers preferentially allocated the biomass to inflorescences, and then the inflorescences preferentially allocated the biomass to seeds throughout reproductive period. Earlier heading tillers had more contribution to *E. excelsus* population adaptation and development in the two peak years of production.

## Introduction

Reproduction is vital to the dynamics and maintenance of plant populations^1–3^. For most herbs, consequences of reproduction, including vegetative propagation and sexual reproduction, is a core issue of plant life-history theory^4–7^. Furthermore, the timing and synchrony of reproduction has received considerable focus in both empirical and theoretical studies as an aspect of life-history theory^8–11^. The timing of reproduction is a strong determinant of offspring viability and reproductive success both in animals^12–15^ and plants^10,16–18^. However, the consequences of reproduction involves synchrony or asynchrony within populations is a matter of debate.

Reproductive synchrony refers to the tendency of individuals to carry out some stage of the reproductive cycle simultaneously with other members of the population^19^. Then reproductive asynchrony refers to an asynchronous tendency. They are two common phenomena occurring among plants^9^. Reproductive synchrony and asynchrony may be adaptive or nonadaptive^11,20,21^. The hypothesis, originally presented by Darling^23^, proposes that reproductive synchrony is an antipredation strategy, which is potentially the most general mechanism in both animals and plants^22,23^. Bronstein *et al*.^24^ consider that for plants with temporally separate sexual phases to outcross, population-level flowering asynchrony is necessary^24^. In previous research, consequences of reproductive asynchrony within plant populations is inconsistent. The study on large *Lupinus lepidus* colonizing population after 1980 eruption of Mount St. Helens indicates the surviving population more asynchronous individuals have more fruits^8^. Reproductive success of non-rewarding *Cypripedium japonicum* benefits from asynchronous flowering^20^. Similarly, a cactus with asynchronous flowering (*Pilosocereus leucocephalus*) shows the flowering phenology is beneficial to its reproductive success^25^. On the contrary, in wheat the synchrony of male and female flowering is a crucial determinant of seed set^10^. Although some studies have evaluated the influence of reproductive asynchrony to population reproduction, the consequences of asynchronous reproduction are still not clear in the peak years of production in a perennial bunchgrass grass.

Phenotypic plasticity of plants is still a central issue of ecological and evolutionary research^26,27^. Phenotypic plasticity in different reproductive stages is of vital significance for plant species to adapt to heterogeneous environments^28,29^, which is vitally important for understanding growth regulation and matter allocation at population level^30^. A study on perennial bunchgrass *Puccinellia tenuiflora* has showed that tiller height, tiller biomass, spike length and spike biomass of the reproductive tillers at early heading, heading, and milky stages increase significantly with the increase of reproductive growth period^31^. The growth and production metrics of annual green foxtail show extreme differences among delayed reproduction time^32^. Annual *Chenopodium glaucum* and *Amaranthus retroflexus* display true plasticity in reproductive allocation with the delay of sowing dates. Furthermore, plant height, crown diameter, and reproductive tissue biomass, and seed production of *C. glaucum and A. retroflexus* increase with delayed reproductive period^3^. However, the effect of asynchronous reproduction on phenotypic plasticity and the change rules of phenotypic traits with the time difference of asynchronous reproduction is not well known.

Based on the heading and flowering asynchrony of *Elymus excelsus* reproductive tillers, all heading reproductive tillers from early to late are regarded as a continuum of heading reproductive tillers^33^. In the present study, in the third and fourth cultivated year when the grass reached a production peak, heading reproductive tillers of *E. excelsus* were marked and measured in five different heading times of two consecutive years. The objectives of the study were in the peak years of *E. excelsus* production: (a) to inquiry the differences of growth, biomass, seed production and reproductive allocation among asynchronous heading reproductive tillers; (b) to reveal the change rules of growth, production and allocation metrics with difference in reproductive period due to asynchronous reproduction; (c) to explore the contribution of asynchronous reproduction to population adaptation. We hypothesize that (1) phenotypic characteristics of reproductive tillers will have markedly differences among asynchronous heading dates, (2) phenotypic characteristics of reproductive tillers will significantly increase with the increase of differences in reproductive period in two sampling years, and (3) reproductive tillers will preferentially allocate biomass to reproductive parts (inflorescences and seeds) throughout heading period. Furthermore, for those cases where we observed asynchrony effects, we tested (4) whether earlier heading tillers will have a more contribution than later heading tillers for future population.

## Results

### Growth and biomass

**Figure 1:**
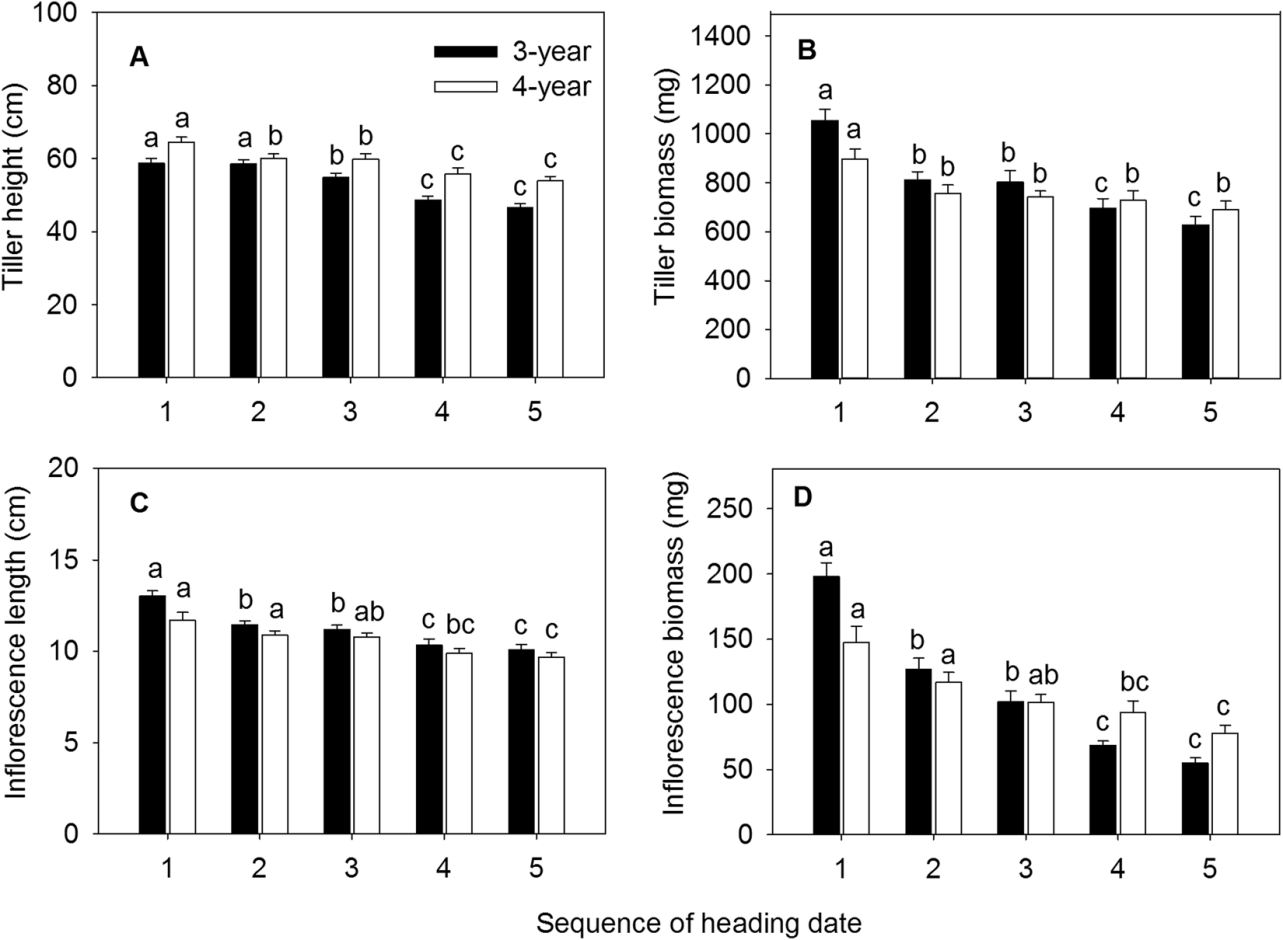
Effect of heading dates on tiller height (**A**), tiller biomass (**B**), inflorescence length (**C**) and inflorescence biomass (**D**) in experimental *Elymus excelsus* populations in two growing years (3-year and 4-year plants). Data are means ± S.E.M. Different letters for the same growing year indicate statistical difference at *P* < 0.05 among heading dates. Five heading dates are July 25, July 29, August 2, August 6 and August 10 of 2009 in 3-year-old plants and July 19, July 23, July 27, July31 and August 4 of 2010 in 4-year-old plants. Five heading dates in each growing year form a heading sequence. Arabic numeral 1, 2, 3, 4 and 5, in turn, represents five heading date in each growing year, respectively.

Tiller height, tiller biomass, inflorescence length, and inflorescence biomass of *E. excelsus* showed a decreasing trend with the delay of heading date over the two years. For 3-year-old reproductive tillers, the height significantly reduced from the third heading date (Aug 2) (Fig. 1A), and biomass, inflorescence length, and inflorescence biomass significantly decreased from the second heading date (Jul 29) (Fig. 1B–D). For 4-year-old reproductive tillers, the height, biomass, and inflorescence biomass significantly reduced from the second heading date (Jul 23) (Fig. 1A,B,D), but inflorescence length began to decrease significantly from the fourth heading date (Jul 31)(Fig. 1C). These results indicated that earlier heading promoted tiller height growth, inflorescence elongation, and tiller biomass accumulation, especially to inflorescence biomass accumulation.

Compared to the last heading date, tiller height, tiller biomass, inflorescence length and inflorescence biomass of the first heading date was 26.5%, 68%, 28.7% and 2.6 times higher in 3-year-old tillers and was 19.7%, 29.6%, 20.6%, 88.8% higher in 4-year-old tillers. These results demonstrated that tiller height and inflorescence length of *E. excelsus* were relatively stable over two years, whereas tiller biomass and inflorescence biomass fluctuated markedly.

### Seed production

**Figure 2:**
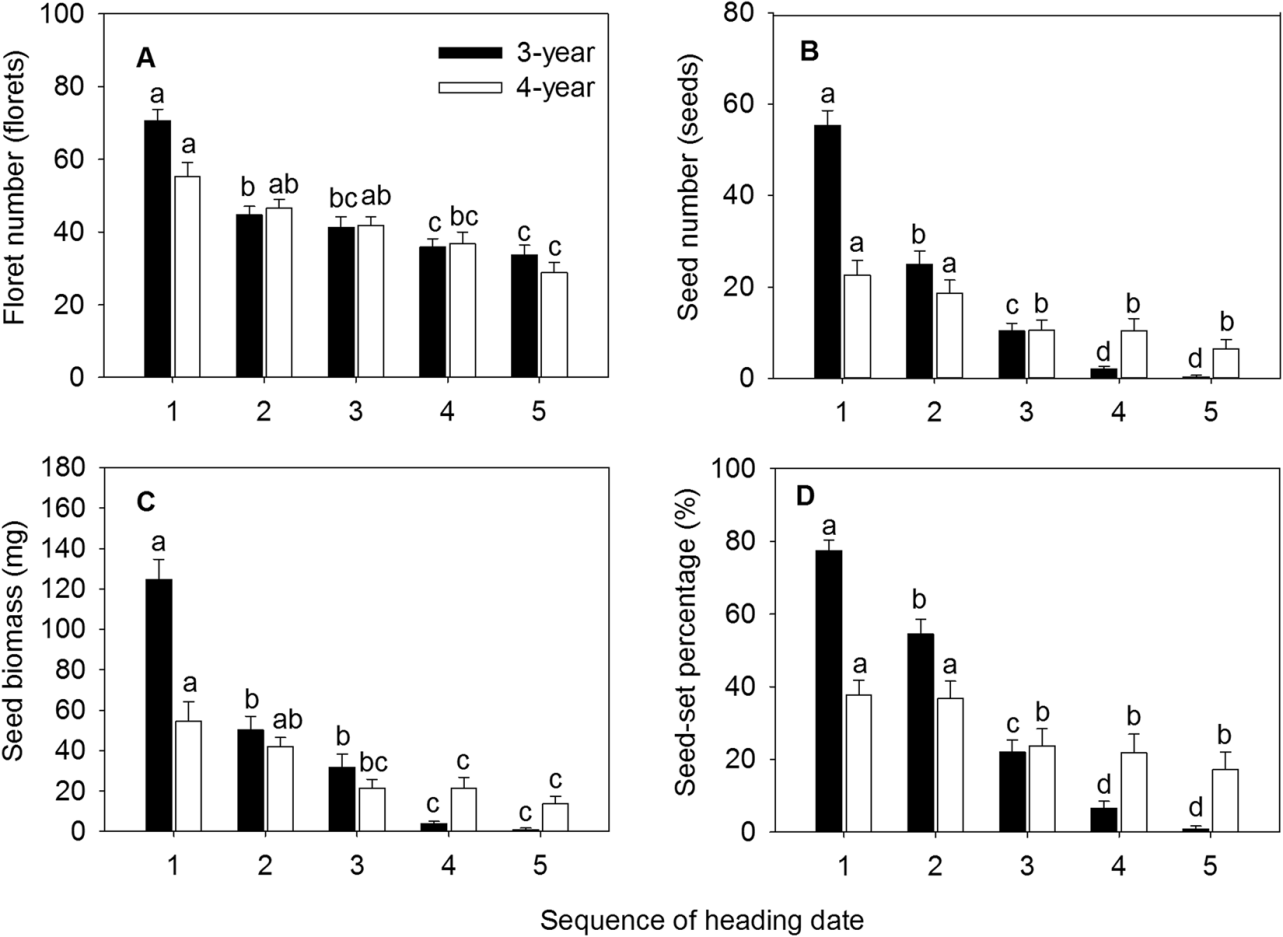
Effect of heading dates on floret number (**A**), seed number (**B**), seed biomass (**C**) and seed-set percentage (**D**) in experimental *Elymus excelsus* populations in two growing years (3-year and 4-year plants). Data are means ± S.E.M. Different letters for the same growing year indicate statistical difference at *P* < 0.05 among heading dates. Five heading dates are July 25, July 29, August 2, August 6 and August 10 of 2009 in 3-year-old plants and July 19, July 23, July 27, July31 and August 4 of 2010 in 4-year-old plants. Five heading dates in each growing year form a heading sequence. Arabic numeral 1, 2, 3, 4 and 5, in turn, represents five heading date in each growing year, respectively.

**Figure 3:**
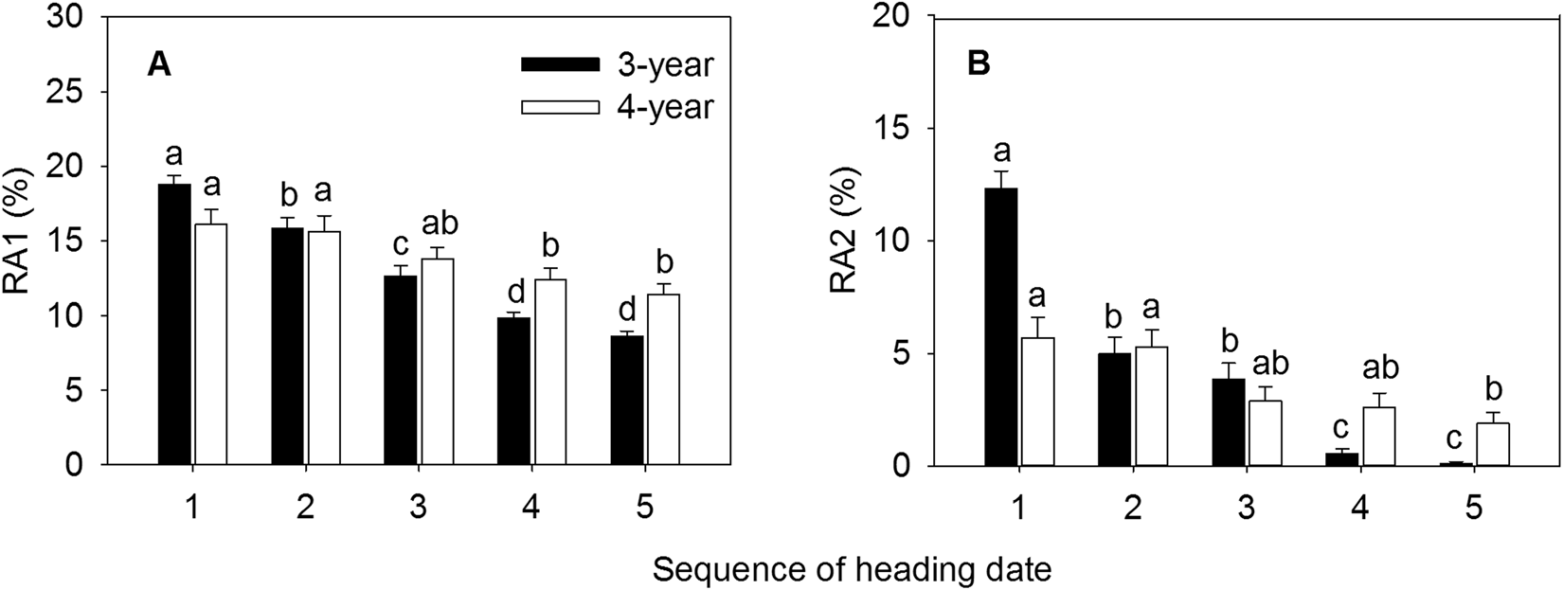
Effect of heading dates on RA1 (**A**) and RA2 (**B**) in experimental *Elymus excelsus* populations in two growing years (3-year and 4-year plants). Data are means ± S.E.M. Different letters for the same growing year indicate statistical difference at *P* < 0.05 among heading dates. Five heading dates are July 25, July 29, August 2, August 6 and August 10 of 2009 in 3-year-old plants and July 19, July 23, July 27, July31 and August 4 of 2010 in 4-year-old plants. Five heading dates in each growing year form a heading sequence. Arabic numeral 1, 2, 3, 4 and 5, in turn, represents five heading date in each growing year, respectively.

Number of florets, number of seeds per spike, seed biomass, and seed-set percentage showed a decreasing trend with the delay of heading date over the two years. For 3-year-old reproductive tillers, number of florets, number of seeds per spike, seed biomass, and setting rate significantly decreased from the second heading date (Jul 29) (Figs 2A–C and 3D). For 4-year-old reproductive tillers, number of florets began to decrease significantly from the fourth heading date (Jul 31) (Fig. 2A), whereas number of seeds per spike, tiller biomass, and setting rate reduced significantly from the third heading date (Jul 27) (Fig. 2B–D). These results indicated that the earlier heading tillers were more beneficial to the formation of florets, seed production, and for improving setting rate.

Number of florets, number of seeds per spike, seed biomass, and setting rate was 1.1 times, 183.0 times, 154.8 times and 85.0 times higher in the first heading date than in the last heading date in 3-year-old tillers and the value was 91.0%, 2.5 times, 1.2 times and 3.0 times higher, respectively, in 4-year-old tillers. These results demonstrated that number of florets in *E. excelsus* population was relatively stable, whereas number of seeds per spike, seed biomass, and setting rate fluctuated a great deal across the two sampling years.

### Reproductive allocation

Biomass allocation to inflorescence (RA1) and biomass allocation to seed (RA2) showed a decreasing trend with the delay of the heading date over two years. RA1 and RA2 of 3-year-old tillers significantly decreased from the second heading date (Jul 23) (Fig. 3A,B). RA1 of 4-year-old tillers began to reduce statistically from the fourth heading date (Jul 31), and RA2 did from the fifth heading date (Aug 4) (Fig. 3A,B). These results indicated that the earlier heading tillers were more beneficial to allocation to inflorescence and seed biomass. Compared to RA1 and RA2 between the first and the last heading date, 3-year-old tillers was 1.2 times and 122.0 times higher, respectively, and 4-year-old tillers was 41.2% and 2.0 times higher, respectively, indicating that RA1 and RA2 of *E. excelsus* population fluctuated wildly during two sampling years.

### Relationships between phenotypic characteristics and reproductive period

**Figure 4:**
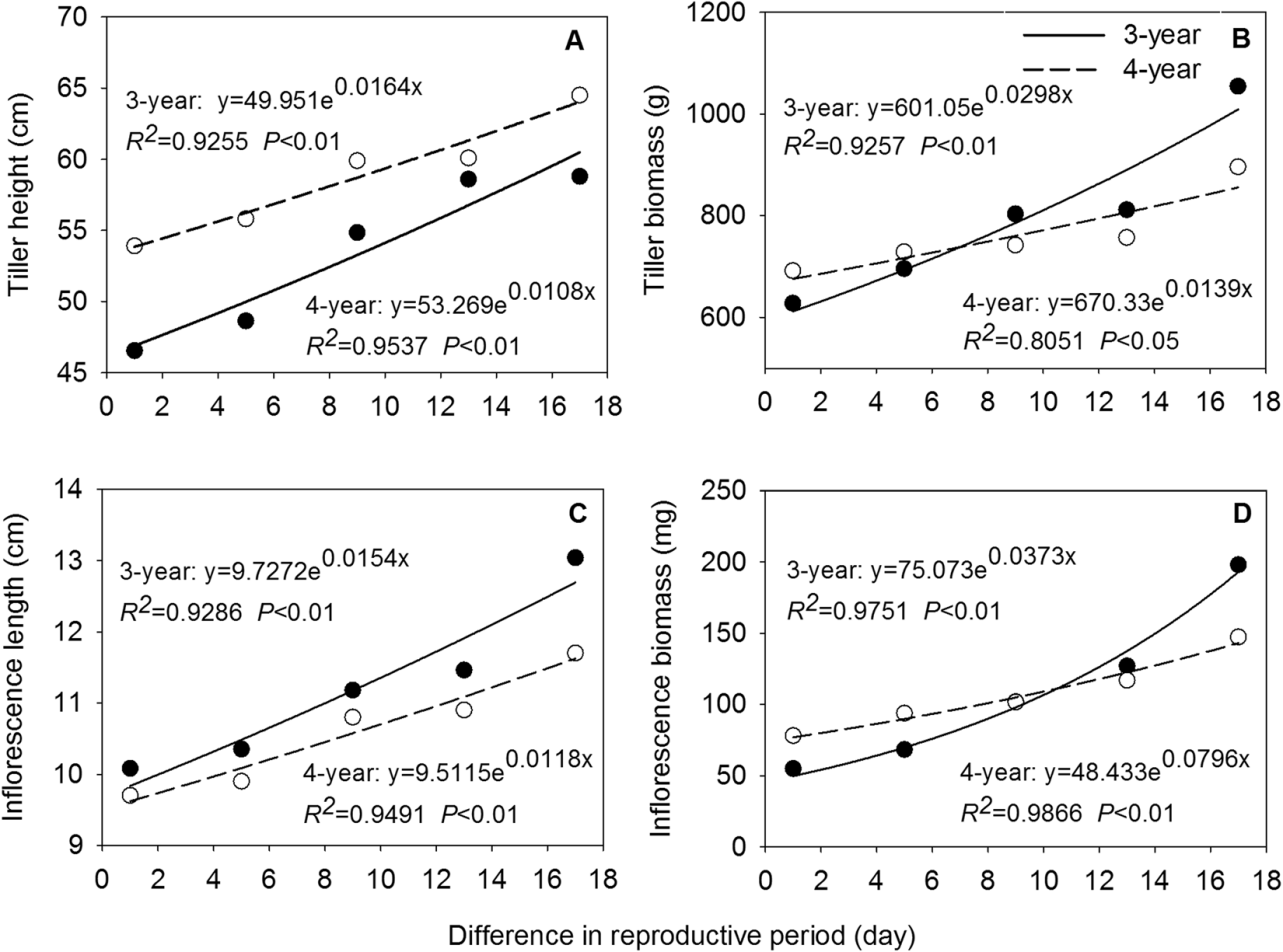
Tiller height (**A**), tiller biomass (**B**), inflorescence length (**C**) and inflorescence biomass (**D**) in relation to the difference in reproductive period (difference between reproductive period of any given heading date and reproductive period of last heading date plus one) in experimental *Elymus excelsus* populations in two growing years (3-year and 4-year plants). The differences are 17, 13, 9, 5, and 1 days from the first to last heading date in 3-year plants of 2009 and 4-year plants of 2010. Solid circles represent observed values of 3-year plants. Hollow circles represents observed values of 4-year plants. Lines are from regression analyses.

**Figure 5:**
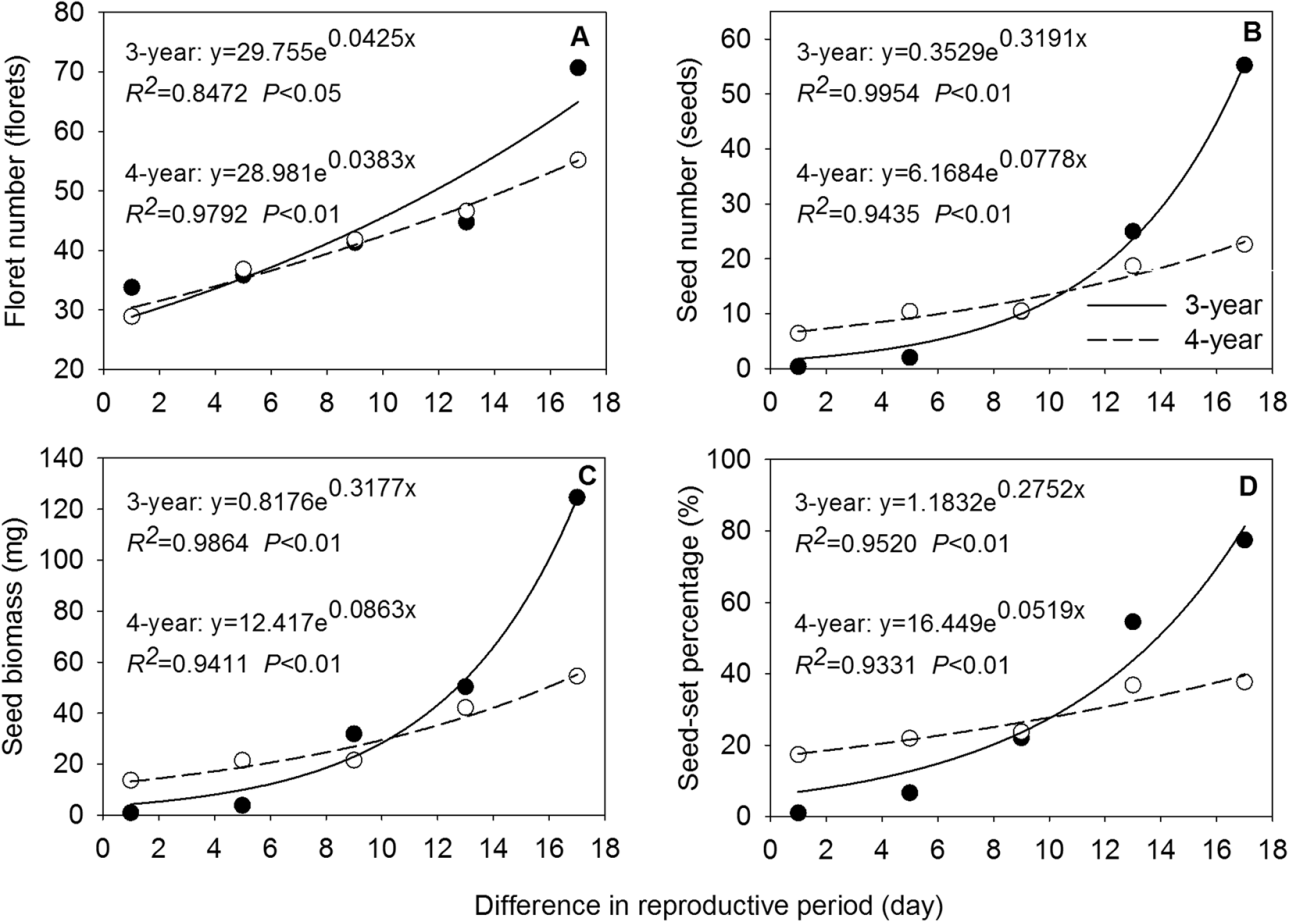
Floret number (**A**), seed number (**B**), seed biomass (**C**) and seed-set percentage (**D**) in relation to the difference in reproductive period (difference between reproductive period of any given heading date and reproductive period of last heading date plus one) in experimental *Elymus excelsus* populations in two growing years (3-year and 4-year plants). The differences are 17, 13, 9, 5, and 1 days from the first to last heading date in 3-year plants of 2009 and 4-year plants of 2010. Solid circles represent observed values of 3-year plants. Hollow circles represents observed values of 4-year plants. Lines are from regression analyses.

**Figure 6:**
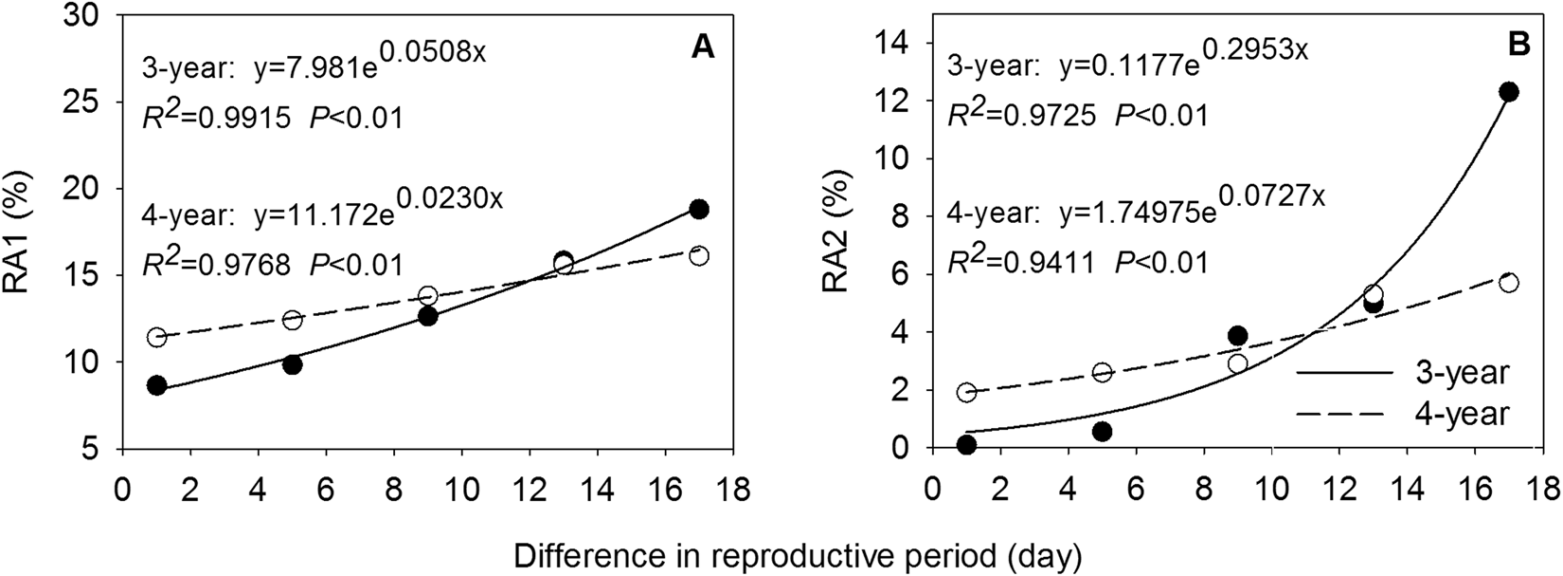
RA1 (**A**) and RA2 (**B**) in relation to the difference in reproductive period (difference between reproductive period of any given heading date and reproductive period of last heading date plus one) in experimental *Elymus excelsus* populations in two growing years (3-year and 4-year plants). The differences are 17, 13, 9, 5, and 1 days from the first to last heading date in 3-year plants of 2009 and 4-year plants of 2010. Solid circles represent observed values of 3-year plants. Hollow circles represents observed values of 4-year plants. Lines are from regression analyses.

With increasing difference in reproductive period across two sampling years, tiller height, inflorescence length, floret number, seed number, tiller biomass, inflorescence biomass, seed biomass, seed-set percentage, RA1, and RA2 of *E. excelsus* population increased significantly by an exponential function (Figs 4, 5 and 6). Except for number of florets in 3-year-old tillers and tiller biomass in 4-year-old tillers (*P* < 0.05) (Figs 4B and 5A), all other relationships were at an extremely significant level (*P* < 0.01) (Figs 4A,C,D, 5B,C,D and 6A,B). Above results showed all ten traits followed the same rules with increasing reproductive period.

## Discussion

Inflorescence of grass is characteristically arranged in spikelets, each having one or more florets. The spikelets are further grouped into panicles or spikes. Due to the differences in formation time of spikelets and florets and the order of nutrient transport, the florets on the same inflorescence do not flower at the same time, so even in a single inflorescence the flowering period is asynchronous, and then resulting in flowering period in population can last for some time^10,34^. For our study material, *E. excelsus*, the heading and flowering time is obviously asynchronous within populations. The difference may be caused mainly by different formation time in reproductive tillers. *E. excelsus* populations have a long vegetative growth period after seed ripening period, approximately from mid-August to early October, in which tiller buds and juvenile tillers can continuously produce in Songnen Plain. In the next year, 85.5% of juvenile tillers develops into winter tillers, and 24.5% of overwintering buds develops into spring tillers^35^. Different growth time in winter and spring tillers results in asynchronous heading and flowering time within populations. In addition, previous results suggest reproductive asynchrony is favored in a temporally variable, coarse-grained environment^36^. A recent theoretical model predicts that whether a population displays reproductive synchrony or asynchrony will depend on the relative scales of intrinsic regulation and environmental disturbance experienced by reproducing individuals^9^. In our study, the precipitation was obviously fluctuant from 2007 to 2010 (2007: 291.7 mm, 2008: 395.3 mm, 2009: 282.2 mm and 2010: 444.7 mm). Phenotypic characteristics such as tiller height, tiller biomass, inflorescence length, inflorescence biomass, seed number, seed biomass were significantly affected by fluctuant precipitation in two sampling years in *E. excelsus* populations, but the change rules of all ten traits with difference in reproductive period were similar in two years, indicating asynchrony reproduction in *E. excelsus* population is determined by intrinsic factor in the two peak years of matter production.

Plasticity response of phenotypic characteristics on reproductive asynchrony is common in plants^20^. In Songnen Plain of Northeast China, as a perennial bunchgrass grass, asynchronous heading of *E. excelsus* populations in natural and cultivated conditions is obvious. Heading date of reproductive tillers in *E. excelsus* populations is approximately from mid-July to early August, and the period lasts for about 30 days as well as forms a continuum of heading reproductive tillers^33^. In our study, asynchronous heading significantly affected phenotypic characteristics of reproductive tillers in experimental *E. excelsus* populations over two growing seasons. Late-heading tillers were shorter and their biomass was lower than early-heading tillers. Moreover, earlier heading plants produced greater inflorescence, with much more floret number, seed number, seed biomass, seed-set percentage, RA1 and RA2 resulting from the longer period of vegetative and reproductive growth (Figs 1C,D, 2 and 3). The results prove our first hypothesis. In the large *Lupinus lepidus* colonizing population, mean flowering date has a direct positive effect on fruit number in all three sampling years, furthermore, in the surviving population more asynchronous individuals have more fruits^8^. Significant effects of asynchronous heading on ten phenotypic characteristics of reproductive tillers in our study suggest a wide variation in the phenotypic plasticity along the sequence of five heading dates over two years. This shows the growth strategies of tillers at sequence of heading period. The earlier the time of flowering is, the easier it is for the plant to occupy space, utilize resources, and get more pollen at the flowering stage and easy to fertilize^37–39^.

Plasticity variation is enormous and allocation strategy is diverse in different reproduction period in plant^39^. In this study, all reproductive tillers from early to late heading were regarded as a continuum^40^. Same exponential and positive relationships between ten quantitative characteristics in reproductive tillers of experimental *E. excelsus* populations with the difference in reproductive period over two years suggest uniform rules of phenotypic characteristics responding to changes of reproductive period in the two peak years of matter production (Figs 4, 5 and 6). The results prove our second hypothesis. The equation parameters of all correlation relationships imply the matter regulation process and allocation strategy in *E. excelsus* population. First, as an accelerating function, identical exponential relationships show an accelerating change process in all ten phenotypic characteristics and the orderly growth and allocation with the prolonged reproductive period. Second, for the increasing rate b value, inflorescence biomass (b = 0.0796) of 3-year-old tillers is 2.7 times higher than tiller biomass (b = 0.0298), and seed biomass (b = 0.3177) is 4.0 times and 10.7 times higher than inflorescence biomass and tiller biomass, respectively. Inflorescence biomass (b = 0.0373) of 4-year-old tillers is 2.7 times higher than tiller biomass (b = 0.01398), and seed biomass (b = 0.0863) is 2.3 times and 6.2 times higher than inflorescence biomass and tiller biomass, respectively. The changes in tiller biomass are regarded as a process of tiller matter production and the changes in seed biomass and inflorescence biomass are regarded as matter accumulation process of reproductive organs^41^. All changes reflect an allocation strategy: reproductive tiller preferentially allocates the biomass to inflorescence, and then the inflorescence preferentially allocates the biomass to seed in the prolonged reproductive period in experimental *E. excelsus* populations, which test our third hypothesis.

Consequences of reproductive asynchrony within plant populations is a matter of debate^8–11^. On the one hand, the timing and synchrony of male and female flowering in wheat is a crucial determinant of seed set^10^. On the other hand, highly synchronous flowering in *Cypripedium japonicum* populations is not advantageous for reproductive success^20^. Reproductive success of non-rewarding *C. japonicum* benefits from asynchronous flowering and low spatial dispersion pattern^20^. *Pilosocereus leucocephalus*, a columnar cactus with asynchronous pulsed flowering, its flowering onset has a significant and positive relationship with fruit set^25^. Furthermore, asynchronous flowering reduces seed predation in a perennial forest herb *Actaea spicata*^42^. The strong intra-tree asynchrony in flowering in a dioecious fig tree, *Ficus hirta*, maintains successfully the fig wasp population within an individual plant^43^. During our study, in the two peak years of *E. excelsus* production, earlier-heading reproductive tillers yield more production matter and higher quantity of seeds in experimental *E. excelsus* populations (Figs 1B and 2B). In 3-year-old reproductive tillers, the mean tiller biomass in first heading date was 68.0% higher than last heading date and the inflorescence biomass was 2.6 times higher. In the last sampling of reproductive tillers, the mean number of seeds per spike was considerably smaller (only 0.3 seeds per tiller) (Fig. 2B) and the seed-set percentage was really lower (only 0.9%) (Fig. 2C), some reproductive tillers did not even set any seed. Accordingly, seed-setting traits and reproductive allocation showed a tremendous difference between the first and the last date heading. The seed number per inflorescence, seed-setting percentage and RA2 in the first heading date was 183.0 times, 85.0 times and 122.0 times higher, respectively, than the last heading date. In the experimental *E. excelsus* populations, the earlier heading reproductive tillers make a more contribution to population survival and development. The results just answer our fourth hypothesis. In contrast, the latest heading tillers plays a certain role in maintaining present populations and its contribution to the population development is very small.

## Conclusions

In an experimental population of perennial bunchgrass *E. excelsus*, asynchronous heading affected significantly on reproductive tiller height, reproductive tiller biomass, inflorescence length, inflorescence biomass, floret number, seed number, seed biomass, seed-set percentage, biomass allocation to inflorescence (RA1) and seed (RA2) and they gradually decreased with the delay of heading date in the two peak years of *E. excelsus* production. With the increase of differences in reproductive period, above ten phenotypic characteristics all increased exponentially at a significant or extremely significant level. Reproductive tillers preferentially allocate the biomass to sexual reproduction parts (inflorescences and seeds) throughout heading period to obtain higher fitness. Contrary to later heading tillers, earlier heading tillers have a more contribution to population adaptation and development in the two peak years of *E. excelsus* matter production.

## Materials and Methods

### Study area

The study was conducted at Grassland Ecology Research Station of Northeast Normal University, Changling, in western Jilin province, China (E123°41′, N44°38′). The area locates in the southern Songnen Plain, which is characterized by a semi-arid, semi-humid and continental monsoonal climate. Mean annual temperature is 4.6 °C–6.4 °C. Average daily temperature ≥ 10 °C is 120–140 days. Annual accumulated temperature is 3000 °C–3500 °C. Mean annual precipitation is 300 mm–450 mm, with the majority between June and September. Annual evaporation is 1200 mm–1400 mm. A frost-free period is about 130–165 days^39^.

### Study species

*Elymus excelsus* is a perennial bunchgrass plant belonging to Gramineae. The plant is a typical winter grass. It generally flowers from its second growing year. In our study area, the natural *E. excelsus* heads in late July and seeds matures from late August. The grass has a wide adaptability to soil. It is an important component of temperate grassland vegetation in the Northern Hemisphere. It is highly palatable to animals and well suited to establishing high production artificial grassland. The plant can reach a production peak in the third and fourth cultivated year^44^. As a native grass with spikes, the seeds mature from top to bottom in turn. Its average setting rate is about 90.8%. The average one thousand-seed weight is 3.2 g in our previous investigation. The germination rate was above 95%.

### Experimental method

*E. excelsus* seeds were collected in the autumn of 2006 from natural grassland near the research station, and stored dry at room temperature until the study was initiated. The experimental field was planted to corn in the previous year and some annual plants were present prior to initiation of the experiment. Soil type is sandy loam. All vegetation was removed before planting. The experimental plots were established in June of 2007 with five plot replications. Each plot had an area of 12 m^2^ (2 m × 6 m) with rows 0.3 m apart and 0.3 m between plants. Seeds were sown by hand and plots were manually irrigated for several days after sowing to ensure seedling emergence. Young seedlings were thinned to one single plant when they uniformly reached a height of 15 cm. No fertilizer was added to the soil. *E. excelsus* plants were not obviously affected by insect pests throughout the study and all other plants were regularly pulled by hand from 2006 to 2010.

The marking and sampling experiments were processed in 2009 and 2010, the two peak years of *E. excelsus* production. In 2009, at the early heading stage of *E. excelsus*, three-year-old reproductive tillers were randomly marked with tags every four days on 25 July, 29 July, 2 August, 6 August and 10 August, respectively. In each plot 10 tillers were marked and in total 50 tillers in five plots. All marked tillers were harvested together at dough stage on 25 August. Similarly, in 2010, four-year-old reproductive tillers were marked with tags every four days on 19 July, 23 July, 27 July, 31 July, 4 August, respectively, at the early heading stage. All marked tillers were harvested together at the dough stage on 31 August.

The early heading stage was defined as the top of inflorescences reaching out approximately 1 cm over the flag leaf sheath^41^. In order to reduce experimental errors, all tags were marked between 8:00 and 10:00 am on the day of heading. When sampling, the aboveground parts of each marked tiller was cut down. All 50 tillers were collected together with same heading date from five plots. Moreover, 30 tillers (n = 30) were selected randomly from same heading date in lab. Tiller height, inflorescence length, number of florets, and seed number were measured. Each tiller was separated into leaves, stems, inflorescence and seeds. Dry biomass including all parts of tiller was determined after oven-drying to a constant weight at 80 °C.

### Data analysis

All statistical analysis were conducted in SPSS 19.0 software package (SPSS Inc., Chicago, IL, USA). The data are reported as the mean ± S.E.M. (standard error of the mean), and they were checked for assumptions of normality using the Kolmogorov-Smirnov test and for homogeneity of variance using Levene’s test. The significance level of differences were at *P* < 0.05 or *P* < 0.01. If both assumptions were met, the differences of variables among five heading dates in each sampling year were subsequently analyzed with One-way ANOVA followed by Tukey’s multiple comparison test. However, the variables that did not meet both of these assumptions were subjected to a log-transformation before analysis with One-way ANOVA. Thus, the differences of phenotypic traits and contribution of asynchronous reproduction to future population were explored at two peak years of production. All ten variables were tiller height, inflorescence length, floret number, seed number, tiller biomass, inflorescence biomass, seed biomass, seed-set percentage (percentage of number of seeds in number of florets), biomass allocation to inflorescence (RA1, percentage of inflorescence biomass in tiller biomass), and biomass allocation to seed (RA2, percentage of seed biomass in tiller biomass).

In order to reveal the change rule of phenotypic traits with differences of reproductive period, above ten variables were regressed on difference in reproductive period using linear, power, exponential, logarithmic and quadric functions in two growing years (3-year-old and 4-year-old plants), respectively. Here difference of reproductive period was defined that the difference between reproductive period of any given heading date and reproductive period of last heading date plus one day. The best fits were chosen to describe these relationships.

### Data availability

The datasets generated during and/or analysed during the current study are available from the corresponding author on reasonable request.
